# Supplementary material for: A large-scale concordance study of toxicity findings across preclinical species and humans for small molecules and biologics
Source: Front Toxicol. 2026 Apr 2;8:1731947. doi: 10.3389/ftox.2026.1731947 (PMC13082757; doi:10.3389/ftox.2026.1731947)
Supplement: Supplementary file 1 [file Presentation1.pdf]

Supplementary Information (SI)

A Large-Scale Concordance Study of Toxicity Findings Across Preclinical Species and Humans for Small Molecules and Biologics

Xin Liu[1,*], Fan Fan[1]

[1]Preclinical Sciences & Translational Safety, Johnson & Johnson

[*]Corresponding Author: email: xliu332@its.jnj.com

Supplementary Table 1: Summary of past studies on translational concordance between animal and human adverse event.

Study ID	Data source	N of drugs	Metrics	Headline findings	Target organs/systems (if reported)
Olson_2000	International Life Sciences Institute's Health and Environmental Sciences Institute (ILSI-HESI)	150	Sensitivity	Overall: 71 % concordance (any animal vs human); 63% non-rodent vs human; 43% rodent vs human	Higher: haematological, gastrointestinal and cardiovascular (> 80 %). Lower: cutaneous (< 35 %)
Tamaki_2013	Pharmaceuticals and Medical Devices Agency (PMDA)	142	Sensitivity	48% of ADRs were predictable	Higher: haematological, infection, ocular, and injection site reactions (~70%). Lower: cardiovascular, neurological and cutaneous (< 30%).
Clark_2015	PharmaPendium(PP)	3815	Likelihood ratios (LRs)	Animal findings for arrhythmia, QT prolongation had higher LR+ values (e.g., LR+ ≈10–11); lack of animal event is not a good predictor of human safety.	Not organ-level
Monticello_2017	The International Consortium for Innovation and Quality (IQ)	182	Predictive values (PVs)	PPV 43%; NPV 86% – absence of toxicity in animals strongly predicts absence in clinic.	Higher: nervous, gastrointestinal for NHP. Lower: not specified.
Clark_2018	PharmaPendium (PP)	3290	Likelihood ratios (LRs)	Organ system level associations are often highly significant but low to modest in LR+ (<10). Endpoint-level signals are strong for QT/arrhythmia and drug-specific antibody. Negative predictivity (LR–) is limited.	Higher: infections, vascular, immune, skin and musculoskeletal (all 4–9).<br>Lower: general disorders, investigations, and pregnancy (all <3).
Giblin_2021	PharmaPendium (PP)	2259	Mutual information (MI), Likelihood ratios (LRs)	188 statistically significant matching preclinical and clinical terms were identified. Strongest examples include drug specific antibody present (LR+ 118), and blood prolactin increased (LR+ 23). Non-identical term analysis was conducted across organ systems.	Not organ-level

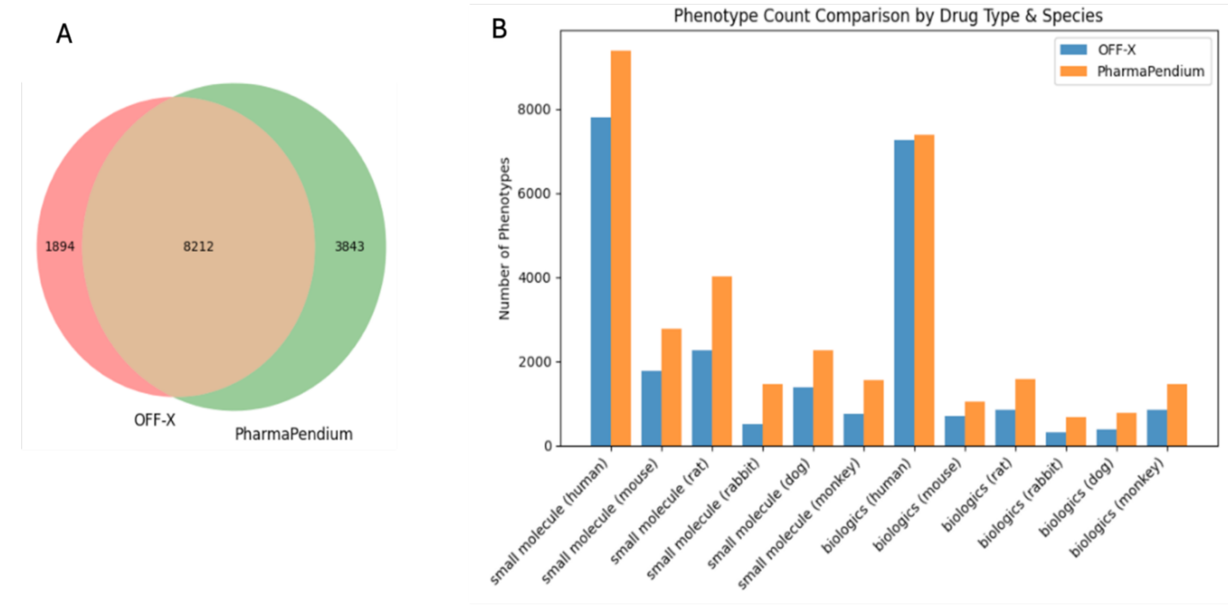

Supplementary Figure 1: Comparison of adverse event phenotypes in OFF-X and PharmaPendium. (A) Venn diagram showing overlapped and unique phenotypes between the two datasets. (B) Number of unique phenotypes by drug type (small molecules, biologics) and species (human, mouse, rat, rabbit, dog, monkey), with OFF-X in blue and PharmaPendium in orange.

Supplementary Table 2: Concordance testing categories and groupings.

**Concordance Type**	**Modalities Included**	**Species Tested**
PT Phenotype – Identical Term	Small Molecule; Biologics; All Modality	Mouse, Rat, Rabbit, Dog, Monkey, All Species
PT Phenotype – Non-Identical Term	Small Molecule; Biologics; All Modality	Mouse, Rat, Rabbit, Dog, Monkey, All Species
PT Phenotype – PK Controlled	Small Molecule; Biologics; All Modality	Mouse, Rat, Rabbit, Dog, Monkey, All Species
SOC Concordance	Small Molecule; Biologics; All Modality	Mouse, Rat, Rabbit, Dog, Monkey, All Species

Supplementary Table 3: Concordance analysis aggregated on SOC level by all Modality and species.

SOC	TP	FN	FP	TN	PPV	NPV	LR+	iLR-	Odds_Ratio	OR_CI_lower	OR_CI_upper	Adjusted_p_value
infections and infestations	1292	3770	60	2443	0.96	0.39	10.65	1.31	13.95	10.72	18.17	1.30E-170
immune system disorders	1620	3794	138	2013	0.92	0.35	4.66	1.34	6.23	5.19	7.47	1.10E-144
psychiatric disorders	1896	3007	580	2082	0.77	0.41	1.77	1.28	2.26	2.03	2.52	1.57E-223
endocrine disorders	1487	2830	306	2942	0.83	0.51	3.66	1.38	5.05	4.42	5.77	7.41E-206
ear and labyrinth disorders	151	2641	71	4702	0.68	0.64	3.64	1.04	3.79	2.84	5.04	8.28E-27
musculoskeletal and connective tissue disorders	2055	3337	172	2001	0.92	0.37	4.81	1.49	7.16	6.07	8.45	1.34E-173
renal and urinary disorders	2324	2455	307	2479	0.88	0.5	4.41	1.73	7.64	6.7	8.72	1.60E-264
cardiac disorders	3089	2878	281	1317	0.92	0.31	2.94	1.71	5.03	4.38	5.78	2.04E-199
reproductive system and breast disorders	1928	1897	494	3246	0.8	0.63	3.82	1.75	6.68	5.96	7.48	9.23E-274
skin and subcutaneous tissue disorders	1991	4040	139	1395	0.93	0.26	3.64	1.36	4.95	4.12	5.94	2.70E-89
respiratory, thoracic and mediastinal disorders	1764	3939	161	1701	0.92	0.3	3.58	1.32	4.73	3.99	5.61	1.27E-93
vascular disorders	1891	4183	134	1357	0.93	0.24	3.46	1.32	4.58	3.8	5.51	5.17E-77
gastrointestinal disorders	3252	3035	194	1084	0.94	0.26	3.41	1.76	5.99	5.1	7.03	5.58E-137
neoplasms benign, malignant and unspecified (incl cysts and polyps)	1327	2014	506	3718	0.72	0.65	3.32	1.46	4.84	4.31	5.44	1.03E-171
hepatobiliary disorders	2199	2352	456	2558	0.83	0.52	3.19	1.64	5.24	4.67	5.89	1.49E-203
eye disorders	1735	2965	345	2520	0.83	0.46	3.07	1.39	4.27	3.76	4.85	2.04E-130
blood and lymphatic system disorders	2166	2977	392	2030	0.85	0.41	2.6	1.45	3.77	3.34	4.25	6.27E-116
pregnancy, puerperium and perinatal conditions	1443	867	1268	3987	0.53	0.82	2.59	2.02	5.23	4.71	5.81	4.15E-217
congenital, familial and genetic disorders	1424	861	1433	3847	0.5	0.82	2.3	1.93	4.44	4	4.93	2.99E-200
metabolism and nutrition disorders	2984	2660	438	1483	0.87	0.36	2.32	1.64	3.8	3.37	4.28	2.49E-119
nervous system disorders	3750	2574	335	906	0.92	0.26	2.2	1.79	3.94	3.44	4.51	1.49E-96
general disorders and administration site conditions	4174	2229	365	797	0.92	0.26	2.08	1.97	4.09	3.58	4.68	6.20E-100
investigations	4787	1297	727	754	0.87	0.37	1.6	2.39	3.83	3.4	4.31	1.03E-104

Supplementary Table 4: Performance metrics for the 25 most prevalent AEs in human, analyzed at PT level for small molecules (left) and biologics(right). Overlapped phenotypes across modalities were bolded, and the ones with high LR+ were highlighted.

SM

AE	TN	FN	FP	TP	PPV	NPV	Positive_Likelihood_Ratio	1/Negative_Likelihood_Ratio	Adjusted_p_value
nausea	1413	3685	22	403	0.95	0.28	6.43	1.09	1.68E-28
**vomiting**	1703	2743	100	977	0.91	0.38	4.74	1.28	1.49E-84
diarrhoea	1850	2686	74	913	0.93	0.41	6.60	1.29	7.49E-104
fatigue	2137	3026	68	292	0.81	0.41	2.85	1.06	5.61E-16
**death**	2068	1515	491	1449	0.75	0.58	2.55	1.58	4.16E-119
decreased appetite	2593	1803	233	894	0.79	0.59	4.02	1.37	5.46E-120
**constipation**	2801	2142	27	553	0.95	0.57	21.49	1.25	8.52E-145
**rash**	2923	2525	4	71	0.95	0.54	20.01	1.03	9.78E-17
gastrointestinal disorder	2782	1706	249	786	0.76	0.62	3.84	1.34	5.26E-108
asthenia	3084	2194	30	215	0.88	0.58	9.26	1.09	1.58E-46
**thrombocytopenia**	3171	2071	40	241	0.86	0.60	8.37	1.10	9.66E-53
anaemia	3166	1976	46	335	0.88	0.62	10.12	1.15	9.95E-82
pruritus	3210	2175	20	118	0.86	0.60	8.31	1.05	8.47E-25
arrhythmia	3154	1816	89	464	0.84	0.63	7.42	1.22	9.94E-103
**neutropenia**	3257	2037	19	210	0.92	0.62	16.11	1.10	6.95E-60
drug interaction	3260	1894	33	336	0.91	0.63	15.04	1.17	5.52E-97
dyspepsia	3371	1926	1	225	1.00	0.64	352.72	1.12	1.98E-91
insomnia	3445	1956	25	97	0.80	0.64	6.56	1.04	1.73E-19
**hypersensitivity**	3488	1978	15	42	0.74	0.64	4.86	1.02	6.80E-07
dry mouth	3498	1922	15	88	0.85	0.65	10.25	1.04	1.54E-22
**oedema peripheral**	3484	1854	71	114	0.62	0.65	2.90	1.04	1.06E-10
palpitations	3531	1893	26	73	0.74	0.65	5.08	1.03	1.55E-12
**seizure**	3241	955	335	992	0.75	0.77	5.44	1.85	3.78E-253
cardiovascular disorder	3429	1316	186	592	0.76	0.72	6.03	1.38	2.12E-143
depression	3503	1432	113	475	0.81	0.71	7.97	1.29	1.12E-129

Bio

AE	TN	FN	FP	TP	PPV	NPV	Positive_Likelihood_Ratio	1/Negative_Likelihood_Ratio	Adjusted_p_value
**vomiting**	599	1344	14	87	0.86	0.31	2.66	1.04	4.29E-02
**death**	806	833	107	298	0.74	0.49	2.25	1.20	1.44E-14
dyspnoea	1018	926	26	74	0.74	0.52	2.97	1.05	5.42E-05
**rash**	1045	909	2	88	0.98	0.53	46.21	1.09	1.28E-23
**neutropenia**	1066	957	1	20	0.95	0.53	21.84	1.02	5.57E-04
**thrombocytopenia**	1083	878	13	70	0.84	0.55	6.23	1.07	5.36E-11
**constipation**	1225	781	6	32	0.84	0.61	8.08	1.04	6.39E-06
infection	1246	648	2	148	0.99	0.66	116.02	1.23	7.36E-59
cardiac failure	1280	680	2	82	0.98	0.65	68.98	1.12	9.25E-32
injection site reaction	1274	656	37	77	0.68	0.66	3.72	1.09	4.95E-10
**seizure**	1296	689	19	40	0.68	0.65	3.80	1.04	1.74E-04
hyperglycaemia	1327	678	2	37	0.95	0.66	34.39	1.05	2.59E-13
leukopenia	1331	689	7	17	0.71	0.66	4.60	1.02	3.56E-02
cardiotoxicity	1345	488	28	183	0.87	0.73	13.37	1.35	2.87E-64
**hypersensitivity**	1371	642	4	27	0.87	0.68	13.87	1.04	2.23E-08
anaphylactic reaction	1375	638	6	25	0.81	0.68	8.68	1.03	2.02E-06
hypotension	1381	628	11	24	0.69	0.69	4.66	1.03	1.08E-03
cardiac disorder	1317	541	85	101	0.54	0.71	2.59	1.11	2.02E-09
tachycardia	1453	558	10	23	0.70	0.72	5.79	1.03	1.28E-04
**oedema peripheral**	1460	547	11	26	0.70	0.73	6.07	1.04	2.63E-05
erythema	1461	487	34	62	0.65	0.75	4.97	1.10	5.31E-13
cardiomyopathy	1450	403	53	138	0.72	0.78	7.23	1.30	3.38E-42
neurotoxicity	1366	360	151	167	0.53	0.79	3.18	1.32	1.25E-26
alopecia	1500	482	28	34	0.55	0.76	3.60	1.05	1.52E-04
dehydration	1506	477	31	30	0.49	0.76	2.93	1.04	3.30E-03

Supplementary Table 5: Terms with the top 25 highest LR+ for small molecules (SM, left) and biologics (right). Overlapped phenotypes across modalities were bolded.

SM

AE	TN	FN	FP	TP	PPV	NPV	Positive_Likelihood_Ratio	1/Negative_Likelihood_Ratio	Adjusted_p_value
newborn persistent pulmonary hypertension	5460	3	0	60	1.00	1.00	inf	21.00	1.44E-136
retrograde ejaculation	5486	11	0	26	1.00	1.00	inf	3.36	3.12E-60
serous retinal detachment	5488	11	0	24	1.00	1.00	inf	3.18	1.14E-55
hypopituitarism	5445	39	0	39	1.00	0.99	inf	2.00	4.76E-76
increased upper airway secretion	5465	30	0	28	1.00	0.99	inf	1.93	3.33E-57
urinary hesitation	5317	156	0	50	1.00	0.97	inf	1.32	1.04E-72
breast pain	5233	244	0	46	1.00	0.96	inf	1.19	6.33E-59
acne	4834	595	0	94	1.00	0.89	inf	1.16	7.02E-86
colitis ischaemic	5335	168	0	20	1.00	0.97	inf	1.12	5.61E-28
clostridium difficile colitis	5180	246	1	96	0.99	0.95	1454.32	1.39	4.15E-118
accommodation disorder	5185	267	1	70	0.99	0.95	1077.21	1.26	1.21E-84
ocular hypertension	5347	104	3	69	0.96	0.98	711.27	1.66	5.17E-104
glaucoma	5135	230	3	155	0.98	0.96	689.52	1.67	1.25E-186
retinal vein occlusion	5310	188	1	24	0.96	0.97	601.25	1.13	1.86E-31
purpura	5096	383	1	43	0.98	0.93	514.49	1.11	1.75E-45
gastrointestinal perforation	4901	495	2	125	0.98	0.91	494.25	1.25	2.67E-118
macular oedema	5228	270	1	24	0.96	0.95	426.86	1.09	6.93E-28
dyspepsia	3371	1926	1	225	1.00	0.64	352.72	1.12	1.98E-91
aptyalism	5357	92	7	67	0.91	0.98	322.90	1.73	3.77E-99
cytopenia	4949	509	2	63	0.97	0.91	272.65	1.12	7.19E-59
drug withdrawal syndrome	4714	629	4	176	0.98	0.88	257.88	1.28	8.77E-146
pulmonary arterial hypertension	5307	184	3	29	0.91	0.97	240.99	1.16	1.39E-36
orthostatic hypotension	4700	695	3	125	0.98	0.87	238.97	1.18	1.58E-100
endometrial cancer	5377	77	11	58	0.84	0.99	210.44	1.75	7.18E-86
prostate cancer	5262	190	7	64	0.90	0.97	189.66	1.34	1.32E-78

Bio

AE	TN	FN	FP	TP	PPV	NPV	Positive_Likelihood_Ratio	1/Negative_Likelihood_Ratio	Adjusted_p_value
medullary thyroid cancer	2009	0	0	35	1.00	1.00	inf	inf	4.16E-74
immune-mediated hepatitis	1883	61	0	100	1.00	0.97	inf	2.64	1.24E-125
immune-mediated dermatitis	1895	58	0	91	1.00	0.97	inf	2.57	1.59E-116
immune-mediated myocarditis	1888	93	0	63	1.00	0.95	inf	1.68	7.00E-75
tuberculosis	1722	258	0	64	1.00	0.87	inf	1.25	8.18E-52
immune-mediated hepatic disorder	1743	266	0	35	1.00	0.87	inf	1.13	1.03E-28
pancreatic carcinoma	1968	40	1	35	0.97	0.98	918.87	1.87	4.32E-51
thyroid cancer	1972	35	2	35	0.95	0.98	493.50	2.00	3.00E-51
cytokine release syndrome	1604	219	3	218	0.99	0.88	267.22	1.99	1.44E-162
bone loss	2016	12	4	12	0.75	0.99	252.50	2.00	7.88E-20
thyroiditis	1852	102	4	86	0.96	0.95	212.26	1.84	4.60E-91
arteriosclerosis	1809	108	6	121	0.95	0.94	159.84	2.11	1.12E-119
enteritis	1829	145	4	66	0.94	0.93	143.34	1.45	5.44E-62
infection	1246	648	2	148	0.99	0.66	116.02	1.23	7.36E-59
embolism	1837	194	1	12	0.92	0.90	107.07	1.06	1.15E-09
cardiac failure	1280	680	2	82	0.98	0.65	68.98	1.12	9.25E-32
skin toxicity	1786	190	7	61	0.90	0.90	62.25	1.32	2.65E-48
autoimmune disorder	1626	251	12	155	0.93	0.87	52.11	1.61	6.71E-103
rash	1045	909	2	88	0.98	0.53	46.21	1.09	1.28E-23
myocarditis	1752	108	26	158	0.86	0.94	40.62	2.43	4.17E-131
hyperglycaemia	1327	678	2	37	0.95	0.66	34.39	1.05	2.59E-13
teratogenicity	1929	11	60	44	0.42	0.99	26.52	4.85	1.67E-49
pain	1197	698	8	141	0.95	0.63	25.31	1.19	6.21E-45
graft versus host disease	1673	258	19	94	0.83	0.87	23.78	1.35	9.79E-56
bradycardia	1818	203	6	17	0.74	0.90	23.49	1.08	1.65E-10

Supplementary Table 6: AEs with the 25 lowest LR+ for small molecules (SM, left) and biologics (right). Overlapped phenotypes across modalities were bolded.

SM

AE	TN	FN	FP	TP	PPV	NPV	Positive_Likelihood_Ratio	1/Negative_Likelihood_Ratio	Adjusted_p_value
inflammation	4600	392	453	78	0.15	0.92	1.85	1.09	2.17E-04
infertility female	4757	138	588	40	0.06	0.97	2.04	1.15	3.05E-03
colitis	4523	794	150	56	0.27	0.85	2.05	1.04	3.99E-03
developmental delay	4886	129	478	30	0.06	0.97	2.12	1.12	3.79E-02
**cardiac disorder**	3409	1163	486	465	0.49	0.75	2.29	1.23	6.26E-42
bone development abnormal	4875	164	444	40	0.08	0.97	2.35	1.14	1.27E-04
infertility male	4957	328	204	34	0.14	0.94	2.38	1.06	1.94E-03
bone marrow disorder	5018	164	314	27	0.08	0.97	2.40	1.10	4.57E-03
pulmonary oedema	4785	536	157	45	0.22	0.90	2.44	1.05	1.85E-04
cardiomyopathy	4571	554	299	99	0.25	0.89	2.47	1.11	8.34E-12
eyelid ptosis	4826	294	345	58	0.14	0.94	2.47	1.12	4.82E-07
hypokinesia	4661	110	698	54	0.07	0.98	2.53	1.30	4.59E-08
**death**	2068	1515	491	1449	0.75	0.58	2.55	1.58	4.20E-119
myelosuppression	4298	921	190	114	0.38	0.82	2.60	1.08	2.82E-13
blood disorder	4165	1173	104	81	0.44	0.78	2.65	1.04	1.03E-08
hepatotoxicity	3617	1073	418	415	0.50	0.77	2.69	1.24	1.95E-51
neoplasm malignant	4511	716	202	94	0.32	0.86	2.71	1.08	3.40E-12
foetal death	4037	49	1334	103	0.07	0.99	2.73	2.33	4.69E-26
**decreased activity**	4484	93	871	75	0.08	0.98	2.74	1.51	7.92E-15
weight decrease neonatal	4969	56	480	18	0.04	0.99	2.76	1.21	3.81E-03
gastrointestinal inflammation	4996	361	137	29	0.17	0.93	2.79	1.05	1.31E-03
teratogenicity	4389	220	758	156	0.17	0.95	2.82	1.46	4.88E-31
fatigue	2137	3026	68	292	0.81	0.41	2.85	1.06	5.61E-16
hepatic necrosis	4689	337	399	98	0.20	0.93	2.87	1.19	2.70E-17
**neurotoxicity**	4087	810	370	256	0.41	0.83	2.89	1.21	3.02E-38

Bio

AE	TN	FN	FP	TP	PPV	NPV	Positive_Likelihood_Ratio	1/Negative_Likelihood_Ratio	Adjusted_p_value
ataxia	1602	275	163	4	0.02	0.85	0.16	0.92	1.38E-04
nervous system disorder	1294	524	191	35	0.15	0.71	0.49	0.93	3.83E-03
eye inflammation	1686	186	137	35	0.20	0.90	2.11	1.10	2.42E-02
liver disorder	1485	387	107	65	0.38	0.79	2.14	1.09	8.87E-05
**death**	806	833	107	298	0.74	0.49	2.25	1.20	1.44E-14
foetal disorder	1727	47	248	22	0.08	0.97	2.54	1.28	1.28E-03
**cardiac disorder**	1317	541	85	101	0.54	0.71	2.59	1.11	2.02E-09
vomiting	599	1344	14	87	0.86	0.31	2.66	1.04	4.29E-02
dehydration	1506	477	31	30	0.49	0.76	2.93	1.04	3.30E-03
dyspnoea	1018	926	26	74	0.74	0.52	2.97	1.05	5.42E-05
immune-mediated adverse reaction	1436	301	169	138	0.45	0.83	2.99	1.30	3.03E-22
gastrointestinal toxicity	1659	306	49	30	0.38	0.84	3.11	1.07	8.14E-04
**neurotoxicity**	1366	360	151	167	0.53	0.79	3.18	1.32	1.25E-26
lung disorder	1722	185	98	39	0.28	0.90	3.23	1.15	5.83E-07
thrombosis	1647	358	22	17	0.44	0.82	3.44	1.03	2.48E-02
**decreased activity**	1760	16	255	13	0.05	0.99	3.54	1.58	7.31E-03
alopecia	1500	482	28	34	0.55	0.76	3.60	1.05	1.52E-04
injection site reaction	1274	656	37	77	0.68	0.66	3.72	1.09	4.95E-10
eye disorder	1565	423	27	29	0.52	0.79	3.78	1.05	1.88E-04
seizure	1296	689	19	40	0.68	0.65	3.80	1.04	1.74E-04
lymphadenopathy	1776	130	103	35	0.25	0.93	3.87	1.20	1.20E-08
immune-mediated nephritis	1895	54	83	12	0.13	0.97	4.33	1.17	3.79E-03
skin disorder	1597	391	26	30	0.54	0.80	4.45	1.06	7.50E-06
leukopenia	1331	689	7	17	0.71	0.66	4.60	1.02	3.56E-02
hypotension	1381	628	11	24	0.69	0.69	4.66	1.03	1.08E-03

.

Supplementary Table 7: AEs with the 25 highest iLR– for small molecules (SM, left) and biologics (right). Overlapped phenotypes across modalities were bolded.

SM

AE	TN	FN	FP	TP	PPV	NPV	Positive_Likelihood_Ratio	1/Negative_Likelihood_Ratio	Adjusted_p_value
newborn persistent pulmonary hypertension	5460	3	0	60	1.00	1.00	inf	21.00	1.40E-136
**medullary thyroid cancer**	5500	1	3	19	0.86	1.00	1742.62	19.99	6.24E-48
**gastrointestinal hypermotility**	5133	29	29	332	0.92	0.99	163.70	12.38	0.00E+00
waxy flexibility	5375	2	126	20	0.14	1.00	39.69	10.75	3.51E-28
testicular disorder	4701	6	761	55	0.07	1.00	6.47	8.75	1.66E-37
pancreatic disorder	5271	13	149	90	0.38	1.00	31.78	7.71	3.90E-113
carcinoid tumour	5499	4	1	19	0.95	1.00	4543.48	5.75	3.59E-47
hyperphagia	5187	19	231	86	0.27	1.00	19.21	5.29	2.39E-90
psychostimulation	5481	8	0	34	1.00	1.00	inf	5.25	4.86E-79
**testicular atrophy**	5034	9	439	41	0.09	1.00	10.22	5.11	1.29E-33
**congenital musculoskeletal disorder**	4384	31	956	152	0.14	0.99	4.64	4.85	4.02E-76
drug abuse	4995	96	121	311	0.72	0.98	32.31	4.14	0.00E+00
**spleen disorder**	4635	4	872	12	0.01	1.00	4.74	3.37	2.88E-05
retrograde ejaculation	5486	11	0	26	1.00	1.00	inf	3.36	3.12E-60
uterine cancer	5399	25	41	58	0.59	1.00	92.72	3.29	6.21E-88
serous retinal detachment	5488	11	0	24	1.00	1.00	inf	3.18	1.14E-55
foetal disorder	4272	112	856	283	0.25	0.97	4.29	2.94	2.70E-114
dysbiosis	5186	16	294	27	0.08	1.00	11.70	2.54	6.03E-21
foetal death	4037	49	1334	103	0.07	0.99	2.73	2.33	4.69E-26
foetal growth restriction	4603	72	727	121	0.14	0.98	4.60	2.31	3.73E-51
arteritis	5426	35	17	45	0.73	0.99	180.10	2.28	2.80E-73
gastrointestinal hypomotility	4842	213	185	283	0.60	0.96	15.50	2.24	3.84E-204
oxidative stress	5005	43	415	60	0.13	0.99	7.61	2.21	3.20E-36
adrenal disorder	4719	68	638	98	0.13	0.99	4.96	2.15	2.44E-43
gastric haemorrhage	5241	104	56	122	0.69	0.98	51.06	2.15	3.21E-138

Bio

AE	TN	FN	FP	TP	PPV	NPV	Positive_Likelihood_Ratio	1/Negative_Likelihood_Ratio	Adjusted_p_value
**medullary thyroid cancer**	2009	0	0	35	1.00	1.00	inf	inf	4.16E-74
teratogenicity	1929	11	60	44	0.42	0.99	26.52	4.85	1.67E-49
**testicular atrophy**	1978	1	62	3	0.05	1.00	24.68	3.88	1.57E-02
**spleen disorder**	1850	3	182	9	0.05	1.00	8.37	3.64	8.51E-06
foetal damage	1825	33	114	72	0.39	0.98	11.66	2.99	5.04E-54
immune-mediated hepatitis	1883	61	0	100	1.00	0.97	inf	2.64	1.24E-125
immune-mediated dermatitis	1895	58	0	91	1.00	0.97	inf	2.57	1.59E-116
osteosarcoma	2037	2	2	3	0.60	1.00	611.70	2.50	1.55E-05
**gastrointestinal hypermotility**	2031	4	3	6	0.67	1.00	406.80	2.50	5.41E-11
myocarditis	1752	108	26	158	0.86	0.94	40.62	2.43	4.17E-131
arteriosclerosis	1809	108	6	121	0.95	0.94	159.84	2.11	1.12E-119
oedematous pancreatitis	2039	2	1	2	0.67	1.00	1020.00	2.00	2.67E-03
bone sarcoma	2038	2	2	2	0.50	1.00	510.00	2.00	3.81E-03
thyroid cancer	1972	35	2	35	0.95	0.98	493.50	2.00	3.00E-51
bone loss	2016	12	4	12	0.75	0.99	252.50	2.00	7.88E-20
cytokine release syndrome	1604	219	3	218	0.99	0.88	267.22	1.99	1.44E-162
ovarian disorder	1965	8	63	8	0.11	1.00	16.10	1.94	1.93E-06
pancreatic carcinoma	1968	40	1	35	0.97	0.98	918.87	1.87	4.32E-51
**congenital musculoskeletal disorder**	1907	4	129	4	0.03	1.00	7.89	1.87	4.40E-02
thyroiditis	1852	102	4	86	0.96	0.95	212.26	1.84	4.60E-91
miosis	2027	5	8	4	0.33	1.00	113.06	1.79	2.07E-05
bone development abnormal	1959	6	74	5	0.06	1.00	12.49	1.77	5.15E-03
immune-mediated myocarditis	1888	93	0	63	1.00	0.95	inf	1.68	7.00E-75
renal disorder	1552	222	99	171	0.63	0.87	7.26	1.66	8.98E-67
injection site inflammation	1858	57	88	41	0.32	0.97	9.25	1.64	1.23E-23

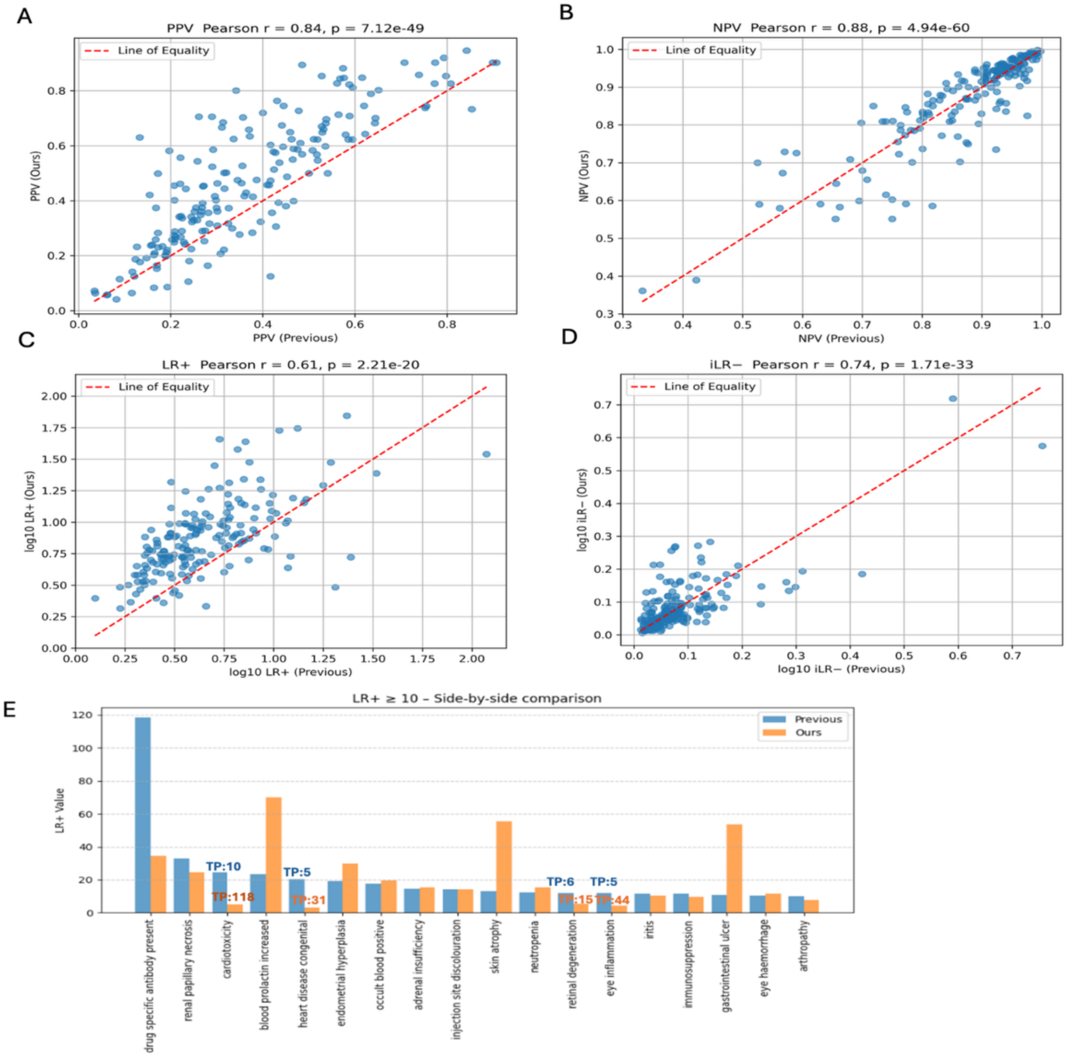

Supplementary Figure 2: Comparison of metrics between our dataset and the previous study (*(Giblin et al., 2021)*). (A) PPV, (B) NPV, (C) LR+ (log10), (D) iLR− (log10) scatter plots comparing our results (Ours) to the previous study (Previous). Each point represents an adverse event aggregated across all modalities and species. Pearson r and p-values quantify correlation.(E) LR+ values for adverse events with LR+ ≥ 10 in the previous study. TP counts are annotated to highlight cases where high LR+ in the previous study were based on few true positives, while our dataset estimates are based on larger case counts.

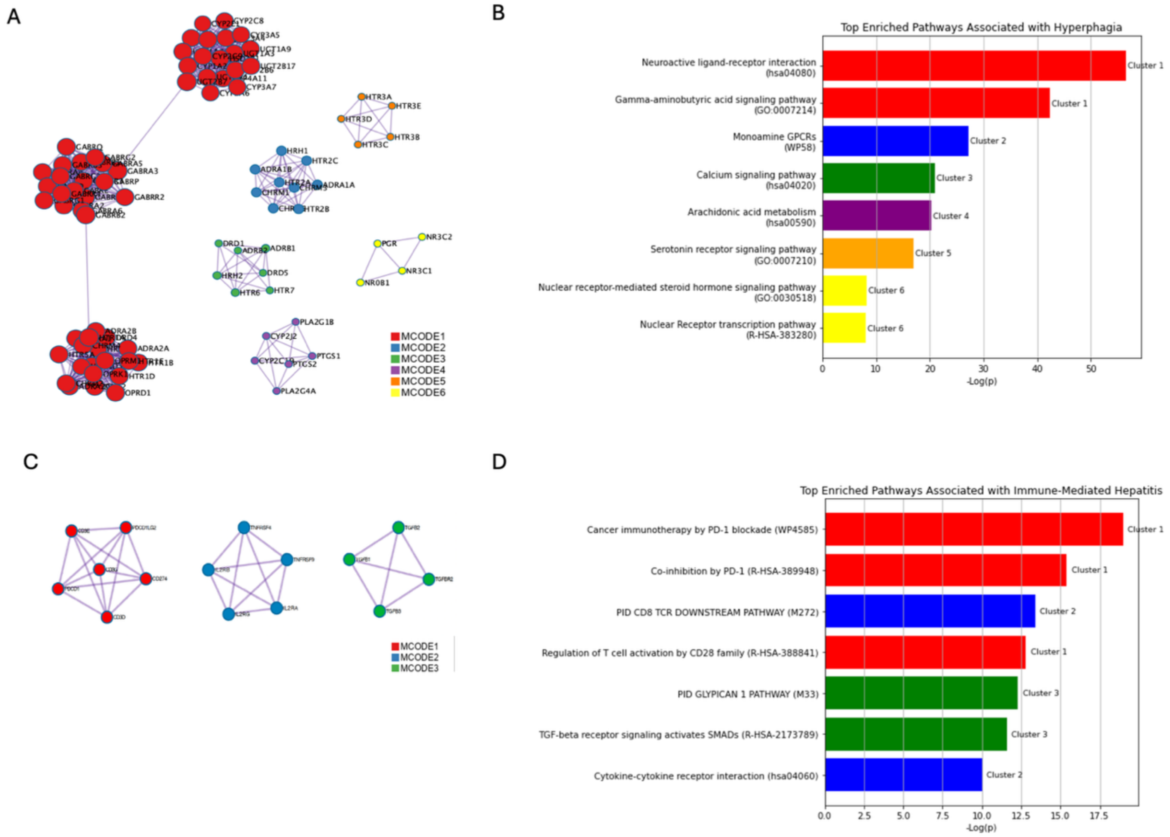

Supplementary Figure 3: Clustering and pathway enrichment analysis of drug targets and off-targets associated with true-positive drugs for hyperphagia and immune-mediated hepatitis.

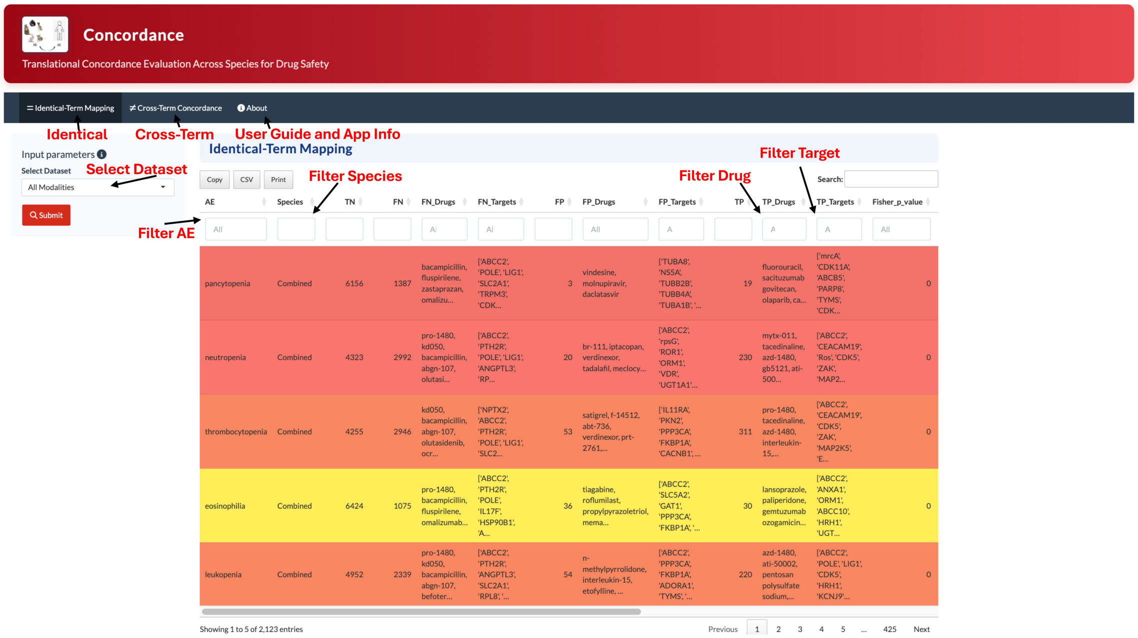

Supplementary Figure 4: Concordance app interface. The top navigation provides access to Identical, Cross-Term Concordance, and About/User Guide. Left controls allow dataset selection; top filters enable narrowing by species, AE, SOC, drug, target, and other columns. The central table lists the concordance results. Export (copy/CSV/print) and pagination controls are available.

Supplementary Table 8: Reproducibility metrics. Exact columns show literal overlap between runs (Jaccard similarity, 0–1). Semantic columns use embedding cosine similarity to measure meaning-level consistency. "LR Avg Abs Diff" reports changes in extracted LR+/PLR values(0 means identical across runs). Higher scores indicate greater stability. N/E = Not Evaluated (as those are within the questions).

Case	Species (Exact)	Species (Semantic)	Targets (Exact)	Targets (Semantic)	Follow-up (Semantic)	LR Avg Abs Diff	Full Answer Semantic
Arrhythmia in dog	**N/E**	**N/E**	0.69	0.92	0.95	0	0.97
Hematuria species selection	1	1	0.44	0.81	0.89	0	0.93
Vomiting translation in rat	**N/E**	**N/E**	0.19	0.74	0.82	0	0.91

Supplementary Table 9: Robustness to prompt variation. The original prompt for each case was compared to paraphrased versions to assess output consistency. "Mean similarity with original" is the average semantic similarity (0–1) between the original prompt's output and each paraphrased prompt's output. "Mean similarity between variations" is the average similarity among paraphrased outputs themselves. "Robustness Drop" is the difference between these two means, with smaller values indicating greater robustness to changes in wording.

Case	Original Prompt	Paraphrased Prompts	Mean Similarity With Original	Mean Similarity Between Variations	Robustness Drop
**Arrhythmia in dog**	I have observed drug induced-arrhythmia in dog, is this adverse event translatable? What are the potential targets and mechanisms involved? What follow-up actions should I take?	1. In a dog study, drug-induced arrhythmia was observed.is this likely to translate to humans? Outline possible biological targets, mechanisms, and recommended next steps.	0.94	0.93	0.01
		2. If dogs develop arrhythmia after drug dosing in preclinical work, how predictive is that for human risk? Include target mechanisms and follow-up actions.			
		3. What is the likelihood that drug-induced arrhythmia in a canine model will occur in humans? Summarize targets, mechanisms, and potential follow-up steps.			
		4. Observed arrhythmia in dogs during preclinical testing, tell me the translatability to humans, underlying pathways, and practical follow-up measures.			
		5. How translatable is drug-induced arrhythmia detected in dogs, and what targets, mechanisms, and next steps should be considered?			
**Hematuria species selection**	Which species would be most suitable for preclinical studies on haematuria? What are the potential targets and mechanisms associated with haematuria findings in selected preclinical species? If I observe adverse effects related to haematuria, what follow-up actions should I consider?	1. For haematuria studies, which preclinical species offer the best predictive value for humans? Identify relevant targets, mechanisms, and follow-up strategies.	0.924	0.918	0.005
		2. What animal species should be used to model haematuria in preclinical studies, and what biological targets and mechanisms are involved? Suggest follow-up if adverse effects occur.			
		3. Choose suitable species for preclinical haematuria research, explaining the targets, mechanisms, and follow-up options.			
		4. In preclinical contexts, which laboratory species are best for haematuria studies? Outline the mechanisms and targets, plus potential next steps if effects are observed.			
		5. When studying haematuria preclinically, what species provide the most translatable data to humans? Include			

		targets, mechanisms, and necessary follow-up actions.			
**Vomiting translation in rat**	I want to know what adverse effects in rat other than vomiting best translate into vomiting in humans for small molecule drugs. Also, what's the potential biological mechanism causing the adverse effect.	1. Which non-vomiting adverse effects in rats are most predictive of vomiting in humans for small molecule drugs? Also, explain the biological mechanisms involved.	0.925	0.927	-0.002
		2. Identify rat adverse effects (excluding vomiting) that best indicate human vomiting risk for small molecule drugs, along with potential mechanisms.			
		3. In rat studies, what adverse events besides vomiting can predict vomiting in humans for small molecules? Provide mechanistic rationale.			
		4. For small molecule drugs, which rat adverse effects other than vomiting translate well into human vomiting responses? Include possible mechanisms.			
		5. Give me rat adverse findings, apart from vomiting ,that strongly translate into human vomiting in the context of small molecule drugs. Also detail relevant biological mechanisms.			
